# Random Field Model Reveals Structure of the Protein Recombinational Landscape

**DOI:** 10.1371/journal.pcbi.1002713

**Published:** 2012-10-04

**Authors:** Philip A. Romero, Frances H. Arnold

**Affiliations:** Division of Chemistry and Chemical Engineering, California Institute of Technology, Pasadena, California, United States of America; University of Sheffield, United Kingdom

## Abstract

We are interested in how intragenic recombination contributes to the evolution of proteins and how this mechanism complements and enhances the diversity generated by random mutation. Experiments have revealed that proteins are highly tolerant to recombination with homologous sequences (mutation by recombination is conservative); more surprisingly, they have also shown that homologous sequence fragments make largely additive contributions to biophysical properties such as stability. Here, we develop a random field model to describe the statistical features of the subset of protein space accessible by recombination, which we refer to as the recombinational landscape. This model shows quantitative agreement with experimental results compiled from eight libraries of proteins that were generated by recombining gene fragments from homologous proteins. The model reveals a recombinational landscape that is highly enriched in functional sequences, with properties dominated by a large-scale additive structure. It also quantifies the relative contributions of parent sequence identity, crossover locations, and protein fold to the tolerance of proteins to recombination. Intragenic recombination explores a unique subset of sequence space that promotes rapid molecular diversification and functional adaptation.

## Author Summary

Mutation and recombination are the primary sources of genetic variation in evolving populations. The relative benefit of these two diversification mechanisms and how they complement each other has been a long-standing question in evolutionary biology. While it is clear what types of genetic diversity these two mechanisms can create, a significant challenge is relating these sequence changes to changes in fitness. The fitness landscape, which describes this mapping from genotype to phenotype, is extraordinarily complex and defined over an incomprehensibly large space of sequences. Here, we develop a model of the landscape that relies not on the details of this mapping, but rather on the statistical relationships between sequences. By studying the expected values of landscape properties, we can gain insights into the structure of the landscape that are independent of the details of how genotype dictates phenotype. We use this random field model to understand how recombination explores a functionally enriched and diverse subset of protein sequence space.

## Introduction

The ubiquity of sex and recombination suggests a significant role in evolution, yet their benefit is still debated [1], [2]. Intragenic recombination events generate chimeric genes, which are believed to make important contributions to allelic diversity in natural populations [3]–[6]. Laboratory experiments clearly demonstrate the benefits of recombining homologous proteins: intragenic recombination generates new proteins that are functionally diverse while still having a high probability of folding properly and functioning [7], [8].

We have developed techniques for the design, construction, and characterization of libraries of chimeric proteins generated by site-directed recombination of homologous sequences [9]–[12]. Briefly, libraries are designed (i.e. crossover sites are selected) to minimize the number of novel residue contacts that are generated upon recombination (we call this number ‘SCHEMA disruption’), which tend to be deleterious to protein function. The sequence fragments chosen this way are then shuffled to generate a combinatorial library of chimeric proteins. The resulting proteins have no random point mutations; all the ‘mutations’ are homologous, that is, to amino acids that already appear in at least one of the parent sequences. These libraries can be used to explore the nature of recombination, without the high levels of random mutations typically found in protein libraries made by DNA shuffling [7] and other, similar methods for homologous recombination *in vitro*.

To date, this laboratory has constructed and tested eight such recombination libraries consisting of chimeric bacterial 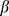-lactamases (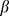
lac13 and 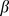
lac), bacterial cytochrome P450s (P450), fungal family 5 cellulases (Cel5), bacterial family 48 cellulases (Cel48), fungal class I cellobiohydrolases (CBHI), fungal class II cellobiohydrolases (CBHII), and human arginases (Arg) (Table 1). Each library, which typically consists of thousands of new sequences, provides a glimpse of the protein fitness landscape that is accessible by recombination, which we refer to as the recombinational landscape. Since every member of the library can be generated by recombining other members, the genetic diversity in these libraries has similarities to that of a sexually reproducing population.

**Table 1:** Summary of eight protein recombination libraries.

library name	protein family	fold class	parent kingdom	sequence length	number of parents 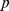	number of crossovers 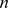	pairwise parent identity (%)	SCHEMA disruption 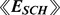	fraction functional 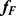	fraction functional 95% CI	additivity 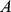	ref.
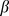 lac13	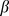 -lactamase	alpha+beta	bacteria	290	2	13	39	91.5	0.007	0.003, 0.022		[34]
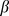 lac	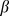 -lactamase	alpha+beta	bacteria	267	3	7	37,42,39	50.2	0.20	0.17, 0.24		[50]
P450	cytochrome P450	all alpha	bacteria	466	3	7	64,65,67	33.4	0.47	0.43, 0.51	0.84	[32]
CBHII	class II cellobiohydrolase	alpha/beta	fungi	361	3	7	65,82,67	15.7	0.48	0.33, 0.63	0.86	[18]
CBHI	class I cellobiohydrolase	all beta	fungi	441	5	7	69,61,72,64,66,73, 81,64,66,70	23.9	0.78**	0.62, 0.90**	0.97	[22]
Cel48	family 48 cellulase	all alpha	bacteria	736	3	7	71,64,65	40.2	0.53	0.43, 0.63	0.73	[21]
Cel5	family 5 cellulase	alpha/beta	fungi	337	3	5	65,31,34	63.1	0.31	0.19, 0.46		unpublished
Arg	arginase	trimeric alpha/beta	animalia	3 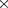 306	2	7	61	23.0	0.50	0.18, 0.81		[20]

The fold class was retrieved from the SCOP structural database [53]. The fraction of functional sequences and additivity were calculated as described in Methods.

** The fraction functional estimates for the CBHI library are significantly biased due to the chimera sampling protocol [22] and are therefore not included in the analysis.

Studies of these libraries have highlighted the enrichment of functional sequences in the recombinational landscape: SCHEMA-designed libraries contain a significant proportion (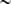
20–50%) of functional sequences, despite having a high average mutation level (i.e. average distance of a chimera sequence from its closest parent). For comparison, random mutation libraries with the same number of mutations are estimated to contain 10–20 orders of magnitude fewer functional sequences [13]–[15]. Whereas random mutations cause the probability a sequence remains functional to decrease exponentially, mutation by recombination always moves towards other functional sequences and is therefore significantly more conservative [16]. For this reason, intragenic recombination effectively explores functional ridges through a protein sequence space that is mostly nonfunctional.

These libraries have also revealed significant variation in thermostability [17], [18] and other properties [19]–[21] within the recombinational landscape. We observed that most of this variation can be explained by additive effects [17], [18], [20]–[22]. That is, the stability, for example, of a chimeric protein can be expressed as the sum of contributions from each of its sequence fragments. This additivity can be used to efficiently engineer highly optimized chimeric proteins for a variety of applications [17], [20], [22], [23]. The additive structure, or lack of epistasis, within the recombinational landscape may provide an abundance of adaptive pathways for natural protein evolution.

We would like to understand the features of the recombinational landscape that contribute to its extreme enrichment in functional sequences and its additive structure. Since the details of the protein recombinational landscape are unknown, we develop a random field model which captures its statistical properties. Random field models are effective at describing statistical features of uncertain, spatially-organized functions, with applications ranging from geostatistics to image analysis [24]–[26]. This versatile class of models has also been used to describe fitness landscapes [27], the best known example being Kauffman's 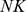-model [28]. Our random field model for the recombinational landscape uses a physics-inspired energy function to describe the sequence-fitness relationship and is parametrized with experimental data. Using this model, we derive approximations for the fraction of functional sequences within a recombination library and the degree of landscape additivity, and we relate these quantities back to experimental observations. We discuss how the structure of the recombinational landscape contributes to the utility of recombination in evolution.

## Results/Discussion

### Random field model of the protein recombinational landscape

**Figure 1:**
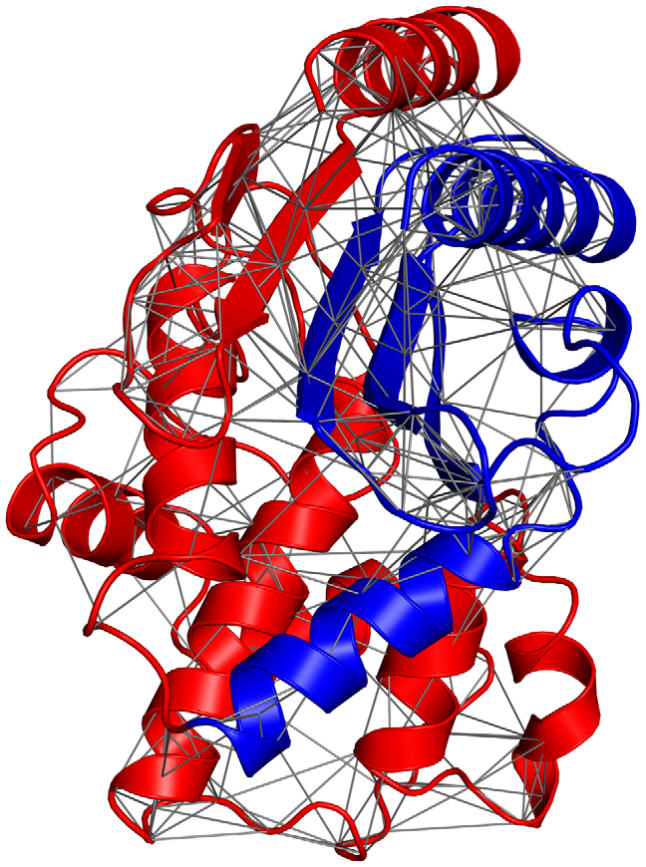
Contact model of protein recombination. When homologous proteins are recombined, structural fragments are acquired from different parents (colored red and blue). Here, lines illustrate contacts between positions that contain residues within 4.5 Å and that are not conserved in the parent sequence alignment. When these nonconserved contacts span structural fragments (i.e., between red and blue) they generate novel (N) interactions that are not observed in either parent. All other contacts, including those between conserved positions (not shown) and those within parental fragments (red-red or blue-blue), provide parental (P) interactions that are found in at least one of the parent structures.

We use a pairwise, residue-level energy function to describe the large number of intramolecular interactions that stabilize protein structures (Figure 1). Such simplified contact potentials have been used in the past for protein folding simulations and structure prediction [29]–[31]. Assuming a fixed structure (set of residue-residue contacts), the energy of any sequence is the sum of energy terms associated with the sequence's specific residue combinations at every pair of contacting residues. For chimeric proteins we distinguish between two types of contacts: parental (P) contacts, which are residue pairs observed in at least one of the parents, and novel (N) contacts, which are not (Figure 1). With this model, the energy of any chimeric protein 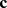 is given by summing the contact energies
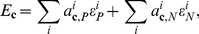
(1)where 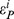 is the energy term associated with parental contact 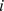, 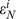 is the energy term associated with novel contact 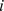, and 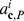 and 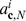 are binary variables which indicate the specific residue pairs for each contact 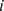 in chimeric protein 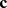. Since the specific energy values of 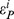 and 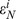 are unknown, we define the independent and identically distributed random numbers 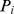 and 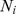, distributed with means and variances
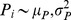
(2)

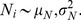
(3)Substituting these random variables into equation 1 defines a random energy function associated with any chimeric protein 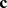


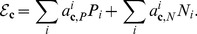
(4)This random energy function is defined over the parental subspace 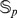, the set of all sequences that can be generated by recombining the parent sequences, which specifies the random field
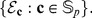
(5)The expected value of the random field at chimeric protein 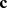 is
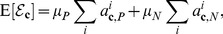
(6)and the covariance between any two sequences is
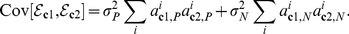
(7)Importantly, this covariance structure expresses how pairs of sequences are related and captures our intuitive notion of protein similarity: proteins with similar sequences have similar structures and therefore similar properties. This random field model provides a statistical description of the recombinational landscape.

To parametrize the random field model, we must determine the mean energy 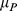 and variance 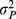 of parental contacts and the equivalent parameters 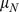 and 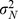 for novel contacts. Using a large binary functional status data set from a library made by recombining three bacterial cytochrome P450 enzyme heme domains [32], these four parameters were estimated by maximizing a marginalized likelihood function (see Methods). If we assume the functional status depends on a sequence's Gibbs free energy difference from the nonfunctional state, these estimated parameters can be interpreted as Gibbs free energy differences in 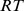 units because the two-state Boltzmann distribution is identical to the logistic likelihood function. As expected, parental contacts are slightly stabilizing (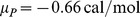), novel contacts are significantly destabilizing (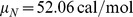), and both classes of contacts show significant variation relative to their means (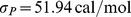 and 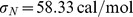). Estimating these parameters on recombination data from other protein families yields qualitatively similar relationships (Figure S1). This is not surprising, considering that most proteins are marginally stable [33] and mutations (novel contacts) tend to be deleterious to protein function [13]–[15]. In the following sections, we use this parametrized random field model to interpret experimental observations from protein recombination libraries.

### Effect of homologous substitutions on protein function

Previously, we compared the effects of random versus homologous amino acid substitutions [16]. Whereas the fraction of functional sequences declines exponentially with increasing random mutations [13], [14], that fraction varies log-parabolically with the number of substitutions taken from another functional parent. For two parents, the log-parabolic behavior appears because accumulating homologous substitutions must eventually convert one functional parent sequence into another functional parent sequence. Random mutagenesis of 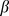-lactamase indicated a probability that a single random mutation will preserve function (neutrality) of 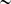
0.54, whereas recombination experiments on the same enzyme indicated the probability a homologous substitution will preserve function (recombinational tolerance) was 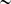
0.79 [16]. A recombinational tolerance significantly larger than the neutrality indicates that homologous substitutions tend to be more conservative than random ones. Here, we evaluate the effects of homologous substitutions using the random field model and compare the results to this previous analysis.

**Figure 2:**
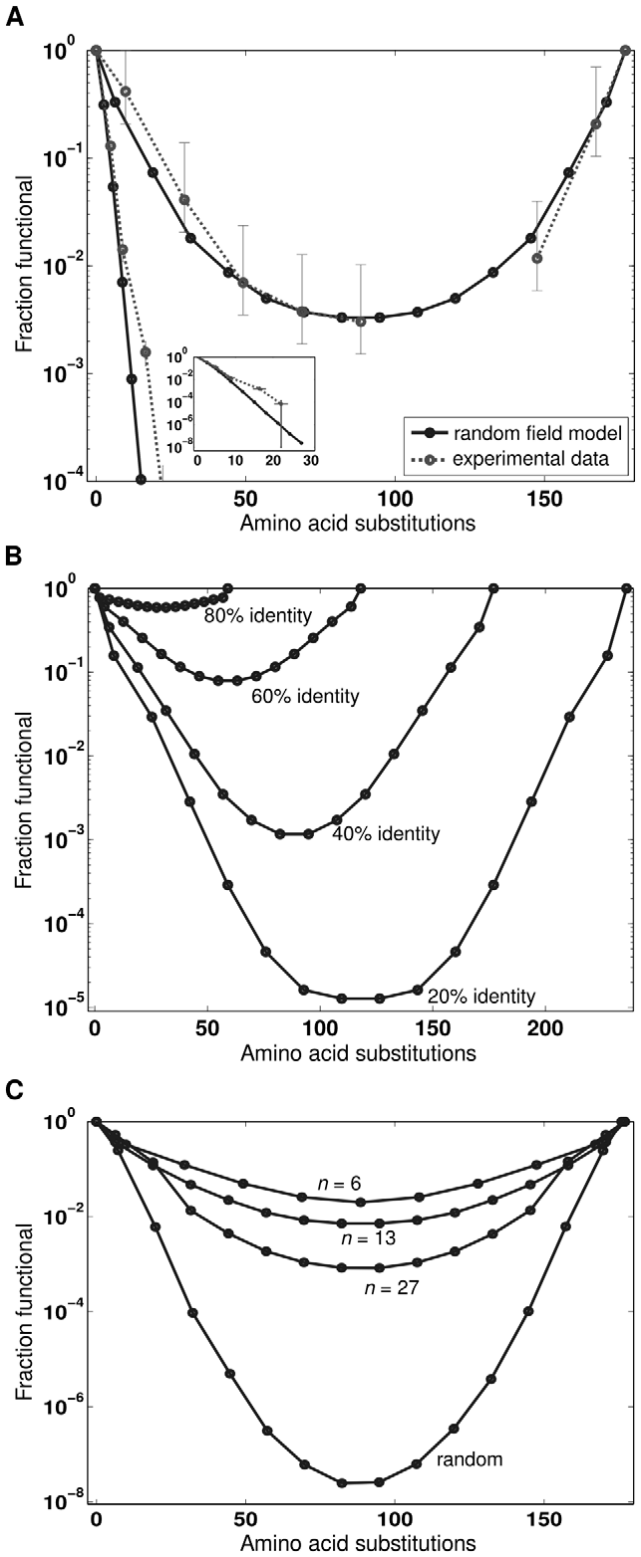
Effect of homologous substitutions on the fraction of functional sequences in a library of chimeric 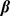-lactamases. (*A*) The random field model agrees well with experimental data on random and homologous substitutions in 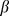-lactamase [16]. The parabolic curve displays the effect of homologous substitutions, and the error bars represent the 95% confidence intervals of the fraction of correctly constructed chimeras (see Methods). The steep exponential curves (and inset) show the effect of random mutations, and the error bars represent one standard error. (*B*) As parent sequence identity decreases, the homologous substitution curves stretch to higher levels of mutation and lower fraction functional. Shown are the substitution curves for the 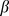
lac13 library (crossover locations and contacts) averaged over 100 random parent sequences with sequence identity ranging from 20–80%. (*C*) As the number of crossovers 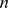 decreases, the homologous substitution curve shifts towards a higher fraction functional. Shown are the substitution curves for the 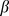
lac13 library (parents and contacts) averaged over 100 random crossover locations with the number of crossovers varying from 6 to 27. The random homologous substitution curve was generated by averaging over 100 randomly sampled sequences at each level of mutation.

Analyzing a library of chimeric 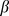-lactamases (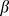
lac13) [34], the probability of functioning for each chimera was estimated by evaluating the logistic function 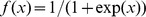 at the expected value of the random field (equation 6). These probabilities were averaged within 15 groups of chimeras binned by their number of homologous substitutions. The same analysis was also performed on simulated random substitutions, where a novel contact was any residue pair not present in the two 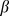-lactamase parents. With two parents, at least 18/19 random mutations will result in non-parental amino acids and therefore novel interactions with any contacting residues. The random field model results show excellent agreement with the experimental results of substitutions generated by recombination and randomly (Figure 2*A*). As observed previously, the fraction of functional sequences undergoes a steep exponential decline with random mutations, while functionality displays a log-parabolic dependence on homologous substitutions.

With the random field model, we can now explore how key recombination parameters, such as parent sequence identity or the number of sequence crossovers, influence the shape of the recombination curve shown in Figure 2*A*. As the sequence identity shared by the parents decreases, the curve stretches to a higher level of mutation (more mutations are possible for a fixed sequence length) and to a lower fraction functional (Figure 2*B*), as was shown previously using lattice protein simulations [16]. Here we see that homologous substitutions from more-distant parents tend to be more deleterious to protein function than substitutions from less-distant proteins. This happens because distant proteins are more likely to have their contact networks composed of different residues, and these networks are therefore less compatible when recombined. We also see that as the number of sequence crossovers decreases, the log-parabolic recombination curve shifts towards a higher fraction functional (Figure 2*C*), necessarily approaching a flat line when there are no crossovers. This happens because each crossover event creates opportunities to generate deleterious interactions. This improvement to the previous analysis allows us to see how recombinational tolerance depends on the number of sequence crossovers. To estimate the effects of homologous amino acid substitutions independent of the number of crossovers, we sampled random homologous substitutions and calculated the average probability of folding at each level of mutation (Figure 2*C*). The effects of random homologous substitutions still follow the log-parabolic curve, although this curve dips over five orders of magnitude lower than the curve obtained from the 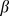-lactamase library experiments [34]. Fitting the log-parabolic equation [16], we estimate the recombinational tolerance of random homologous substitutions to be 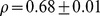. The recombinational tolerance is still significantly greater than the neutrality (0.54), but to a lesser degree than previously estimated.

The only difference between random homologous substitutions and those generated by recombination (Figure 2*C*) is how the mutations are distributed throughout the sequence and structure. Random homologous substitutions are distributed uniformly throughout the sequence, while those generated by recombination occur in contiguous stretches of sequence. By making mutations in groups, recombination preserves many local interactions. From this analysis, we propose an updated model for the conservative nature of intragenic recombination which includes contributions from homologous substitutions (as shown previously) as well as groups of coevolved residues that vary simultaneously. The latter effect is expected to be particularly important in natural evolution, where the number of intragenic crossover events per generation is likely to be small.

It is interesting that the random field model for the recombinational landscape also works reasonably well to describe the effects of random mutations. Random mutations frequently result in a non-parental amino acid and therefore cause deleterious novel interactions with all contacting residues. This simplified model recapitulates the exponential decline in functional sequences that was observed upon random mutagenesis of 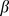-lactamase (Figure 2*A*) and in other mutational studies [13]–[15]. In addition, this model trivially captures the well-known fact that surface mutations tend to be less deleterious than mutations in the protein core, because core residues tend to have many more contacts. With a single model to explain the effects of both random and homologous substitutions, we can understand their differences in terms of residue contacts. The number of deleterious contacts generated by a homologous substitution is less than or equal to the number generated by a random mutation at the same position, with equality rarely being achieved. This is consistent with the explanation that homologous substitutions are conservative because they have been previously selected to be compatible with the protein fold [16].

### Effect of intragenic recombination across protein families

The factors that determine a particular protein family's tolerance to recombination are unknown. Table 1 reports the fraction of functional sequences in eight recombination libraries, representing protein families of different functions, sizes, and fold classes. Seven of these libraries were designed with the intent of maximizing the fraction of functional sequences, yet there is significant variation (2–3 fold) in this fraction between libraries. While some of this variation is likely due to experimental differences in classifying functional versus nonfunctional sequences for different enzymes, we expect a significant proportion of this variation to arise from differences in parent fold, parent sequence identity, and the specific crossover locations chosen in the library design. Using the random field model, we derive an approximation for the expected value of the fraction of functional sequences in a recombination library and use this to understand how these factors contribute to a protein's tolerance to recombination.

Consider a recombination library 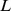 generated by recombining sequence fragments from 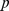 parental sequences at 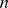 crossover sites. We refer to the sequence fragments between crossover sites as ‘blocks’; therefore the library is composed of 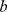 sequence blocks (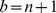). All sequence fragments in these libraries are roughly the same length, and therefore, with the random field model, we can assume that each fragment's energetic contribution is an independently and identically distributed Gaussian random variable. With this assumption, the distribution of sequence energies within recombination library 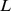 is Gaussian and can be described by its mean
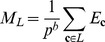
(8)and variance
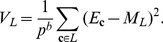
(9)The fraction of functional sequences within library 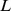 is given by evaluating the Gaussian cumulative distribution function at zero
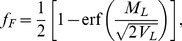
(10)where 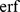 is the error function.

Since the specific energy terms that shape the recombinational landscape are unknown, we use the random field model to calculate the expected value of the fraction of functional sequences by integrating over all possible energy terms 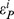 and 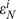. The expected value of the library mean is given by
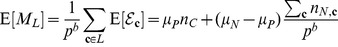
(11)where 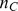 is the total number of contacts and 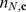 is the number of novel contacts in chimera 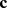. The expected value of the library variance is given by
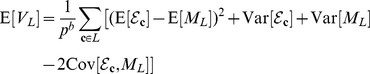
(12)More specific details of 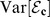, 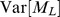, and 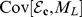 are given in Text S1. With these two expectations, the expected value of the fraction of functional sequences can be approximated with a leading-order Taylor series expansion about 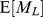 and 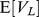


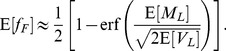
(13)


**Figure 3:**
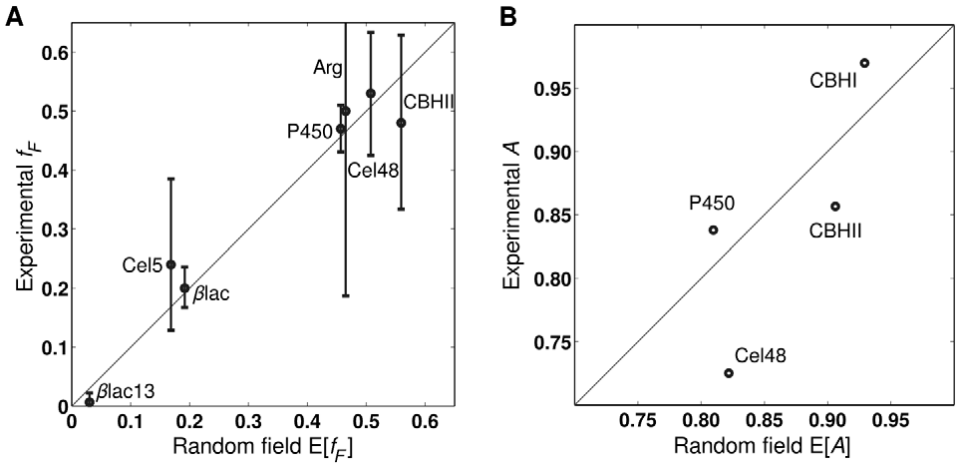
Comparison between library properties and their expected values within the random field model. Note diagonal lines represent 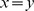. (*A*) The random field's expected fraction of functional sequences shows quantitative agreement with experimental results (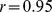 with 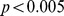). Error bars represent the binomial 95% confidence intervals calculated using the Clopper-Pearson method [51]. (*B*) The expected additivity agrees well with experimentally determined values (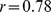 with 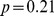). While the small data set limits the statistical significance of this correlation, all 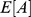
s are large and within the ranges that are observed experimentally.

The expected value of the fraction of functional sequences within a library 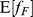 shows quantitative agreement with the experimentally determined values, as shown in Figure 3*A*. With the random field model, both parental and novel contacts contribute to the distribution of sequence energies within a recombination library and therefore to the fraction of functional sequences. The deleterious novel contacts dictate the mean energy of the library (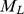), while parental contacts, which typically outnumber novel contacts 50–100-fold, dominate the variance (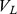). This suggests recombination events can cause loss of function by two independent mechanisms: (1) by introducing new deleterious interactions between sequence fragments, or (2) by introducing sequence fragments that already contain deleterious interactions.

To better understand the variation in the fraction of functional sequences in the different recombination libraries, we sampled random libraries, calculated 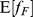, and estimated the contribution from protein fold, specific breakpoints, and parent sequence identity. For each protein fold, we sampled 100 random two-parent sequence alignments with sequence identity ranging from 10–90%, and for each of these alignments we sampled 100 random 7-crossover libraries, for a total of 90,000 libraries. A three-way analysis of variance shows the protein fold (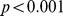), specific breakpoints (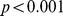), and parent sequence identity (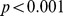) all make significant contributions to the 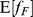. Estimating the variance components, we find parent sequence identity to be the main determinant of 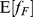 (contributing 92% of the variance), followed by specific crossover locations (4%), and protein fold (2%). This strong dependence on parent sequence identity is the result of the approximately exponential increase in the number of (deleterious) novel contacts as parent sequences diverge.

Interestingly, the parent sequence identity also dictates the mechanism of chimeric protein inactivation. When the parent sequence identity is low, most of the nonfunctional chimeric proteins are the result of new deleterious interactions between sequence fragments. However, when the parent sequence identity is high, nonfunctional sequences are usually the result of inheriting sequence fragments which already contain deleterious interactions. This is consistent with the observation of high mutual information between a chimeric protein's functional status and its number of novel contacts for the 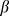-lactamase library (low parent sequence identity) and the low mutual information observed for the P450 library (high parent sequence identity) [35]. In the 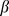-lactamase library, the number of new interactions between fragments (novel contacts) is predictive of the functional status of chimeras. However, in the P450 library, the number of novel contacts is not predictive, suggesting other mechanisms must be responsible for chimera inactivation (i.e. acquisition of deleterious sequence fragments).

### Additive structure of the recombinational landscape

Perhaps the most surprising finding from protein recombination experiments has been the additive structure of the recombinational landscape [17], [20], [22], [23], [36]. Linear models are able to explain a majority of variation in stability as well as some other properties, suggesting that sequence elements make largely independent, additive contributions to a protein's overall properties. In quantitative genetics, this is referred to as additive genetic variance, which according to Fisher's fundamental theorem of natural selection determines a population's response to selection [37], [38]. Additive landscapes are easier for evolving populations to climb because they are not stymied by rugged, epistatic features. This additivity has been especially useful for engineering optimized chimeric proteins in the laboratory, because a small sampling of sequences provides sufficient information to make accurate predictions across the entire library [17], [22], [23]. Here, we use the random field model to understand the origin of the additive structure within the recombinational landscape.

Within the recombination library 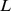 described in the previous section, the total variance can be partitioned into additive and epistatic components (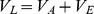). We define the landscape's additivity 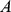 as the fraction of the total variance that is explained by additive effects
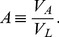
(14)This dimensionless quantity, which ranges from 0 to 1, describes the smoothness of the landscape and is inversely related to the landscape ‘ruggedness’ defined in [39]. For four of the recombination libraries, there are sufficient data to calculate the additivity of the thermostability landscape (see Methods). These results are presented in Table 1.

The additive variation can be understood by considering how individual mutations contribute to variation in the library. A mutation that occurs at a position with a fixed structural context, such as a mutation within a structural fragment inherited from one parent or a mutation surrounded by conserved positions, will always have the same effect throughout the library and therefore contributes entirely to additive variation. However, a mutation can have different effects in different sequences if it occurs at a position whose environment varies. The effects of these mutations can only be expressed with an epistatic (non-additive) model, but their additive contribution can be found by averaging their effects over all structural environments within the library. An additive energy function can be defined by accounting for purely additive and averaged epistatic effects (Text S1). This additive energy can be used to calculate the expected value of a library's additive variance 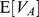 using the same equations as the total variance (previous section). With this, the expected value of the additivity can be approximated with a Taylor series expansion about 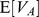 and 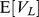


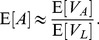
(15)


The expected value of the landscape additivity 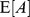 shows close agreement with the experimentally determined values (Figure 3*B*). While the correlation is not statistically significant, due to the limited data, all the 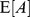
s are large and within the experimentally observed ranges. In addition, the four uncharacterized libraries also have large expected additivities (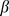
lac13 = 0.44, 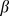
lac = 0.67, Cel5 = 0.65, Arg = 0.82), suggesting this additive structure within the recombinational landscape may be quite general. Despite being generated by a purely pairwise energy function, which is by definition epistatic, a majority of the variation within these recombination libraries can be explained by additive effects. This surprising result can be attributed to two factors: sequence conservation among the parents and the partitioning of interactions into structural modules. Epistatic interactions that are conserved among the parents will not contribute to the variation of any property within the library, and those interactions involving one conserved position will only contribute to additive variation. Epistatic interactions that are partitioned into structural modules will vary together, and therefore contribute to only additive variation. Of the thousands of contacting residues within a chimeric protein, only a small fraction (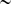
5%) actually contribute to epistatic variation.

The additivity exhibited by the random field model does not hold for chimeric proteins that adopt alternate structures (as described by a contact map). For example, nonfunctional sequences, which account for a significant proportion of chimeras, will clearly not display additivity in properties involving protein function. For many properties, such as thermostability (retention of function at elevated temperatures), where we have observed additivity, the experimental measurements require the chimeras be enzymatically active, which greatly increases the likelihood that they will adopt the same or very similar structures. The subset of sequences that adopt the same structure is referred to as a neutral network [40], [41], and this may define the domain of additivity within the recombinational landscape.

### Summary and conclusions

By using a statistical description of the protein recombinational landscape, we can study the behavior of an astronomical number of sequences–insight which could not be obtained experimentally or even by analyzing homology-based structural models. A probabilistic contact potential was used to specify the mean energy of individual chimeric proteins and how the energy of any sequence is expected to co-vary with others (equations 6 and 7), defining a multivariate probability distribution over all sequences accessible by recombination. While this random field model provides little information about specific sequences, it does reveal the large-scale structure of the recombinational landscape, which we used here to interpret experimental results from past recombination libraries. Within this random field, the expected values of various library properties show excellent agreement with experimental values across multiple protein families. This striking correspondence may arise because a library's properties depend on a large number of interactions, and the cumulative effects of these interactions converge toward the expected value.

The random field model was used to study the enrichment of functional sequences within the recombinational landscape. As shown previously, we found the tolerance of proteins to recombination to be influenced by the conservative effects of homologous substitutions, which have been previously selected to be compatible with the protein fold [16]. However, a more significant contribution comes from groups of coevolved residues varying together. This is especially relevant for understanding natural evolution, where the number of crossover events is relatively low. Evaluating the random field model across protein families, we found parent sequence identity to be the primary determinant for tolerance to recombination, while the specific crossover locations and parent fold make statistically significant, but minor contributions.

Using the random field model, we explored the origins of the additive structure of the recombinational landscape. Both sequence conservation among the parents and the partitioning of epistatic interactions into structural modules make significant contributions to this additivity. The results presented here are for a random field that describes a protein's free energy difference between the functional and non-functional states, which is closely related to protein stability. However, this additivity is generally true for any landscape that is generated from local interactions (including higher order), because sequence conservation and structural partitioning will still be present. This suggests the additivity may apply to numerous biophysical quantities such as binding affinity or substrate specificity.

Previous studies of protein fitness landscapes have highlighted the abundance of nonfunctional sequences [42], [43] and neutral sequence changes [13], [14], [44], suggesting a surface which is mostly flat and filled with holes [45]. In contrast to this full landscape, the recombinational landscape contains orders of magnitude fewer ‘holes’ (non-functional sequences). The functional variation displayed within recombination libraries reveals the large-scale structure of the recombinational landscape, which arises from the cumulative effects of multiple mutations. In addition, most of this functional variation can be explained by additive effects, and additive variation determines a population's response to selection [37], [38] These results were observed in SCHEMA-designed libraries, which tend to be optimized for both functional sequences and additivity. This emphasizes the evolutionary preference for some crossover sites over others, which could explain the presence of recombination hotspots in natural genes [6], [46], [47]. The picture of the recombinational landscape that emerges from the random field model is a surface enriched in functional sequences, which can display locally-epistatic behavior but still has an overall additive structure.

The evolutionary benefit of intragenic recombination may arise because mutation and recombination effectively traverse different landscapes [48]. While climbing the landscape by point mutations, evolution encounters a large number of nonfunctional sequences in addition to epistatic landscape features. In contrast, recombination explores sequences which are much more likely to be functional, in a landscape with an abundance of adaptive pathways. Recombination can provide faster adaptation than point mutation because it generates functional sequences with a large number of substitutions. Recombination may also find sequences that are inaccessible by adaptive point mutation, by simultaneously incorporating multiple coupled mutations, essentially ‘jumping over’ epistatic landscape features. A similar effect was recently described for recombination at the genome level [49], where the authors describe how landscapes arising from high epistasis within genes and no epistasis between genes strongly favors recombination. Running simulations on these ‘modular’ landscapes, the authors found recombination to provide an efficient route to genotypes that were inaccessible by point mutation.

Intragenic recombination is a powerful molecular diversification mechanism. The ubiquity of intragenic recombination in nature and experimental evidence from protein recombination libraries show that it provides distinct advantages over point mutation. In naturally evolving populations, these two genetic variation mechanisms work together. Mutation provides new diversity, while recombination efficiently sorts through the large combinatorial space of existing diversity. A better understanding of how to balance mutation and recombination could assist in engineering highly optimized proteins.

## Methods

### Compiling the chimeric protein data set

Since multiple structures have been solved for each protein family tested, we decided to use all available structures to generate the residue-residue contact map. The contact map for each library was determined by identifying all protein chains within the Protein Data Bank that share at least 50% sequence identity with any parent. Also included were three unpublished P450 structures, for a total of 88 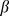
lac13, 173 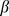
lac, 91 P450, 39 CBHI, 24 CBHII, 6 Cel5, 21 Cel48, and 143 arginase chains. For each chain, a residue pair was considered contacting if they contained any heavy atoms within 4.5 Å. The final contact map for each library is composed of residue pairs that are contacting in more than 50% of all chains. We believe this ‘averaged’ residue-reside contact map should provide a more complete description of the protein family's fold, but the use of any single structure does not change the results presented above.

The number of functional and nonfunctional chimeric proteins was retrieved from previously published results: 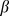
lac13 [34], 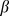
lac [50], P450 [32], CBHI [22], CBHII [23], Cel5 (unpublished data), Cel48 [21], Arg [20]. The fraction of functional chimeras was estimated using maximum likelihood, and 95% confidence intervals were calculated using the Clopper-Pearson method [51]. We could not accurately estimate the fraction of functional sequences for the CBHI library due to the extreme bias in chimera sampling [22]. The results from the 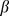
lac13 library were reanalyzed to account for library construction errors (see below).

The additivity of the P450, CBHI, CBHII, and Cel48 libraries was calculated using published thermostability data [17], [21]–[23]. For each library, a block-based linear regression model [17] was parametrized on all the available data. The resulting predictions are unbiased, so the total variance can be partitioned into explained and residual components. The ratio of the explained variance to total variance is the additivity 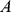, and in this case is identical to the regression model's coefficient of determination 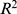.

### Estimation of parental and novel contact parameters

Given a data set which maps contact information to binary functional status, we want to estimate the mean energy 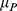 and variance 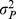 of parental contacts and the mean energy 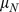 and variance 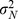 for novel contacts. The true energy terms 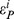 and 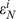 can be integrated out to give the marginalized likelihood function
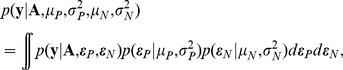
(16)where 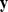 is the binary functional status and for notational simplicity all parental energy terms 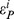 are combined in the vector 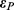, all novel energy terms 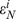 are combined in the vector 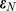, and all binary indicator variables (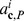 and 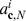) are combined into the matrix 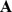. The mean and variance of parental and novel contacts can be estimated by maximizing this marginalized likelihood function.

Since 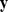 is composed of binary data, we assume that it is generated from a Bernoulli process whose proportion is determined by the energy of a sequence. With this assumption, the first term in the integrand is given by the logistic likelihood function
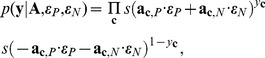
(17)where 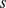 is the logistic sigmoid function given by 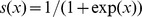, 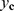 is the binary functional status of chimera 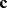, 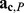 is a vector containing all 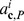, and 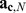 is a vector containing all 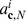. Assuming the energy components are Gaussian distributed, the second and third terms of the integrand are given by multivariate Gaussian distributions. Since the integral in equation 16 is analytically intractable, we can approximate it using Laplace's method [52]. First we approximate the integrand with a multivariate Gaussian about a stationary point and then we evaluate the Gaussian integral to yield
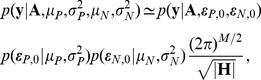
(18)where 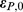 and 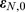 are the stationary points, 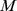 is the fixed number of contacts, and 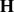 is the Hessian matrix evaluated at the stationary points. The stationary points were found using Newton's method and the marginalized likelihood function was maximized using the Nelder-Mead method.

### Reanalyzing 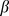-lactamase data to account for library construction errors

The 13-crossover 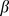-lactamase library (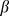
lac13) was assembled from synthetic fragments and had a significant number of construction errors [34]. Sequencing of unselected chimeric genes found 9 of 13 to have frame shift mutations [16], which almost certainly result in inactive proteins. Since a majority of frame shifts are incorporated at the PCR step during library construction, it is likely these errors are present throughout all constructed chimeras [11]. The maximum likelihood estimate for the proportion of correctly constructed chimeras is 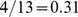, with 95% confidence intervals between 0.09 and 0.61 using the Clopper-Pearson interval [51]. The sequencing data indicate there may be one to three sequence fragments (chimera blocks) that contain frameshift mutations. Assuming all frame shifts cause inactivation and exhaustive library coverage (over twelvefold sampling), the fraction of functional chimeras can be estimated as the number of functional chimeras divided by the number of correctly constructed chimeras. With these assumptions, we estimate the fraction of functional sequences to be 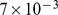 with 95% confidence intervals between 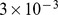 and 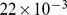 The same modification can be performed on chimeras binned by the number of homologous substitutions (Figure 2*A*) because the construction errors display little relation to the level of mutation.

## Supporting Information

Figure S1
**Estimation of contact parameters on other recombination libraries.** The parental and novel contact parameters (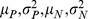) were estimated on four binary functional status data sets. The number of sequences in each data set are indicated in the plot titles. The estimated parameters are reported as the mean 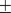 one standard deviation, and the associated Gaussian probability density functions are plotted. The two largest data sets (P450 and 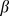-lactamase) give very similar parameter estimates, while all data sets provide the same qualitative relationships among parameters. Within all four parameter sets, we see the mean of parental contacts is slightly favorable and novel contacts are significantly deleterious. The means of these two distributions are separated by approximately one standard deviation, indicating it is relatively common for parental contacts to be as deleterious as novel contacts, and vice versa.(TIFF)

Text S1
**Derivation of a library's expected variance and a chimera's additive energy component.** A detailed description of how the expected value of a library's variance 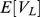 and the additive component of a chimera's energy 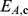 are calculated.(PDF)

## References

[1] Barton NH, Charlesworth B (1998) Why sex and recombination? Science 281: 1986–1990. 9748151

[2] Otto SP, Lenormand T (2002) Resolving the paradox of sex and recombination. Nat Rev Genet 3: 252–261. 11967550 10.1038/nrg761

[3] Watt WB (1972) Intragenic recombination as a source of population genetic variability. Amer Nat 106: 737–753.

[4] Strobeck C, Morgan K (1978) The Effect of Intragenic Recombination on the Number of Alleles in a Finite Population. Genetics 88: 829–844. 17248821 10.1093/genetics/88.4.829 PMC1213820

[5] Freeling M (1978) Allelic Variation at the Level of Intragenic Recombination. Genetics 89: 211–224. 17248830 10.1093/genetics/89.1.211 PMC1213829

[6] De Silva E, Kelley LA, Stumpf MPH (2004) The extent and importance of intragenic recombination. Hum Genomics 1: 410–420. 15606996 10.1186/1479-7364-1-6-410 PMC3500195

[7] Crameri A, Raillard SA, Bermudez E, Stemmer WPC (1998) DNA shuffling of a family of genes from diverse species accelerates directed evolution. Nature 391: 288–291. 9440693 10.1038/34663

[8] Carbone M, Arnold FH (2007) Engineering by homologous recombination: exploring sequence and function within a conserved fold. Curr Opin Struct Biol 17: 454–459. 17884462 10.1016/j.sbi.2007.08.005

[9] Voigt CA, Martinez C, Wang ZG, Mayo SL, Arnold FH (2002) Protein building blocks preserved by recombination. Nat Struct Biol 9: 553–558. 12042875 10.1038/nsb805

[10] Endelman JB, Silberg JJ, Wang ZG, Arnold FH (2004) Site-directed protein recombination as a shortest-path problem. Protein Eng Des Sel 17: 589–594. 15331774 10.1093/protein/gzh067

[11] Farrow MF, Arnold FH (2010) Combinatorial recombination of gene fragments to construct a library of chimeras. Curr Prot Prot Sci 26: 2.1–2.20. 10.1002/0471140864.ps2602s61 20814931

[12] Heinzelman P, Romero PA, Arnold FH (2012) Efficient Sampling of SCHEMA Chimera Families for Identification of Useful Sequence Elements. Method Enzymol In press. 10.1016/B978-0-12-394292-0.00016-3 23422438

[13] Guo HH, Choe J, Loeb LA (2004) Protein tolerance to random amino acid change. Proc Natl Acad Sci U S A 101: 9205–9210. 15197260 10.1073/pnas.0403255101 PMC438954

[14] Bloom JD, Silberg JJ, Wilke CO, Drummond DA, Adami C, et al (2005) Thermo-dynamic prediction of protein neutrality. Proc Natl Acad Sci U S A 102: 606–611. 15644440 10.1073/pnas.0406744102 PMC545518

[15] Bershtein S, Segal M, Bekerman R, Tokuriki N, Tawfik DS (2006) Robustness-epistasis link shapes the fitness landscape of a randomly drifting protein. Nature 444: 929–932. 17122770 10.1038/nature05385

[16] Drummond DA, Silberg JJ, Meyer MM, Wilke CO, Arnold FH (2005) On the conservative nature of intragenic recombination. Proc Natl Acad Sci U S A 102: 5380–5385. 15809422 10.1073/pnas.0500729102 PMC556249

[17] Li Y, Drummond DA, Sawayama AM, Snow CD, Bloom JD, et al (2007) A diverse family of thermostable cytochrome P450s created by recombination of stabilizing fragments. Nat Biotechnol 25: 1051–1056. 17721510 10.1038/nbt1333

[18] Heinzelman P, Snow CD, Wu I, Nguyen C, Villalobos A, et al (2009) A family of thermostable fungal cellulases created by structure-guided recombination. Proc Natl Acad Sci U S A 106: 5610–5615. 19307582 10.1073/pnas.0901417106 PMC2667002

[19] Landwehr M, Carbone M, Otey CR, Li Y, Arnold FH (2007) Diversification of catalytic function in a synthetic family of chimeric cytochrome p450s. Chem Biol 14: 269–278. 17379142 10.1016/j.chembiol.2007.01.009 PMC1991292

[20] Romero PA, Stone E, Lamb C, Chantranupong L, Krause A, et al (2012) SCHEMA-Designed Variants of Human Arginase I and II Reveal Sequence Elements Important to Stability and Catalysis. ACS Synth Biol 1: 221–228. 22737599 10.1021/sb300014t PMC3378063

[21] Smith MA, Rentmeister A, Snow CD, Wu T, Farrow MF, et al (2012) A diverse set of family 48 bacterial cellulases created by structure-guided recombination. FEBS J In press. 10.1111/febs.12032 23075376

[22] Heinzelman P, Komor R, Kannan A, Romero PA, Yu X, et al (2010) Efficient screening of fungal cellobiohydrolase class I enzymes for thermostabilizing sequence blocks by SCHEMA structure-guided recombination. Protein Eng Des Sel 23: 871–880. 20847102 10.1093/protein/gzq063

[23] Heinzelman P, Snow CD, Smith MA, Yu X, Kannan A, et al (2009) SCHEMA recombination of a fungal cellulase uncovers a single mutation that contributes markedly to stability. J Biol Chem 284: 26229–26233. 19625252 10.1074/jbc.C109.034058 PMC2785310

[24] Adler RJ (1981) The Geometry of Random Fields. 1st edition. Chichester: Wiley & Sons.

[25] Stein ML (1999) Interpolation of Spatial Data: Some Theory for Kriging. 1st edition. New York: Springer. 247 p.

[26] Li SZ (2009) Markov Random Field Modeling in Image Analysis. 3rd edition. London: Springer. 362 p.

[27] Stadler PF, Happel R (1999) Random Field Models For Fitness Landscapes. J Math Biol 38: 435–478.

[28] Kauffman SA, Levin SA (1987) Towards a general theory of adaptive walks on rugged landscapes. J Theor Biol 128: 11–45. 3431131 10.1016/s0022-5193(87)80029-2

[29] Miyazawa S, Jernigan RL (1985) Estimation of effective interresidue contact energies from protein crystal structures: quasi-chemical approximation. Macromolecules 18: 534–552.

[30] Clementi C, Vendruscolo M, Maritan A, Domany E (1999) Folding Lennard-Jones proteins by a contact potential. Proteins 37: 544–553. 10651270 10.1002/(sici)1097-0134(19991201)37:4<544::aid-prot5>3.0.co;2-7

[31] Vendruscolo M, Najmanovich R, Domany E (2000) Can a pairwise contact potential stabilize native protein folds against decoys obtained by threading? Proteins 38: 134–148. 10656261 10.1002/(sici)1097-0134(20000201)38:2<134::aid-prot3>3.0.co;2-a

[32] Otey CR, Landwehr M, Endelman JB, Hiraga K, Bloom JD, et al (2006) Structure-Guided Recombination Creates an Artificial Family of Cytochromes P450. PLoS Biol 4: e112. 16594730 10.1371/journal.pbio.0040112 PMC1431580

[33] Taverna DM, Goldstein RA (2002) Why are proteins marginally stable? Proteins 46: 105–109. 11746707 10.1002/prot.10016

[34] Meyer MM, Silberg JJ, Voigt CA, Endelman JB, Mayo SL, et al (2003) Library analysis of SCHEMA-guided protein recombination. Protein Sci 12: 1686–1693. 12876318 10.1110/ps.0306603 PMC2323955

[35] Endelman JB (2005) Design and analysis of combinatorial protein libraries created by site-directed recombination [PhD dissertation]. Pasadena (California): Department of Bioengineering, California Institute of Technology. 122 p.

[36] LeMaster DM, Hernández G (2005) Additivity in both thermodynamic stability and thermal transition temperature for rubredoxin chimeras via hybrid native partitioning. Structure 13: 1153–1163. 16084387 10.1016/j.str.2005.05.007

[37] Fisher RA (1930) The Genetical Theory of Natural Selection. Oxford: Clarendon.

[38] Charlesworth B, Charlesworth D (2010) Elements of evolutionary genetics. Roberts and Co. Publishers, 734 p.

[39] Carneiro M, Hartl DL (2010) Adaptive landscapes and protein evolution. Proc Natl Acad Sci U S A 107: 1747–1751. 19805125 10.1073/pnas.0906192106 PMC2868285

[40] Bornberg-Bauer E (1997) How are model protein structures distributed in sequence space? Biophys J 73: 2393–2403. 9370433 10.1016/S0006-3495(97)78268-7 PMC1181141

[41] Xia Y, Levitt M (2004) Simulating protein evolution in sequence and structure space. Curr Opin Struct Biol 14: 202–207. 15093835 10.1016/j.sbi.2004.03.001

[42] Keefe AD, Szostak JW (2001) Functional proteins from a random-sequence library. Nature 410: 715–718. 11287961 10.1038/35070613 PMC4476321

[43] Axe DD (2004) Estimating the prevalence of protein sequences adopting functional enzyme folds. J Mol Biol 341: 1295–1315. 15321723 10.1016/j.jmb.2004.06.058

[44] Bloom JD, Lu Z, Chen D, Raval A, Venturelli OS, et al (2007) Evolution favors protein mutational robustness in sufficiently large populations. BMC Biol 5: 29. 17640347 10.1186/1741-7007-5-29 PMC1995189

[45] Gavrilets S (2004) Fitness Landscapes and the Origin of Species. Princeton, NJ: Princeton University Press. 476 p.

[46] Yip SP, Lovegrove JU, Rana NA, Hopkinson DA, Whitehouse DB (1999) Mapping recombination hotspots in human phosphoglucomutase (PGM1). Hum Mol Gen 8: 1699–1706. 10441333 10.1093/hmg/8.9.1699

[47] McBee AD, Wegner DJ, Carlson CS, Wambach JA, Yang P, et al (2008) Recombination as a mechanism for sporadic mutation in the surfactant protein-C gene. Pediatr Pulm 43: 443–450. 10.1002/ppul.20782 PMC2765708 18383112

[48] Watson RA, Weinreich D, Wakeley J (2006) Effects of intra-gene fitness interactions on the benefit of sexual recombination. Biochem Soc Trans 34: 560–561. 16856860 10.1042/BST0340560

[49] Watson RA, Weinreich DM, Wakeley J (2011) Genome structure and the benefit of sex. Evolution 65: 523–536. 21029076 10.1111/j.1558-5646.2010.01144.x

[50] Meyer MM, Hochrein L, Arnold FH (2006) Structure-guided SCHEMA recombination of distantly related beta-lactamases. Protein Eng Des Sel 19: 563–570. 17090554 10.1093/protein/gzl045

[51] Clopper CJ, Pearson ES (1934) The use of confidence or fiducial limits illustrated in the case of the binomial. Biometrika 26: 404–413.

[52] Tierney L, Kadane JB (1986) Accurate approximations for posterior moments and marginal densities. J Amer Statist Assoc 81: 82–86.

[53] Murzin AG, Brenner SE, Hubbard T, Chothia C (1995) SCOP: a structural classification of proteins database for the investigation of sequences and structures. J Mol Biol 247: 536–40. 7723011 10.1006/jmbi.1995.0159

