# Supplementary figures and images for: Random Field Model Reveals Structure of the Protein Recombinational Landscape
Source: PLoS Comput Biol. 2012 Oct 4;8(10):e1002713. doi: 10.1371/journal.pcbi.1002713 (PMC3464211; doi:10.1371/journal.pcbi.1002713)

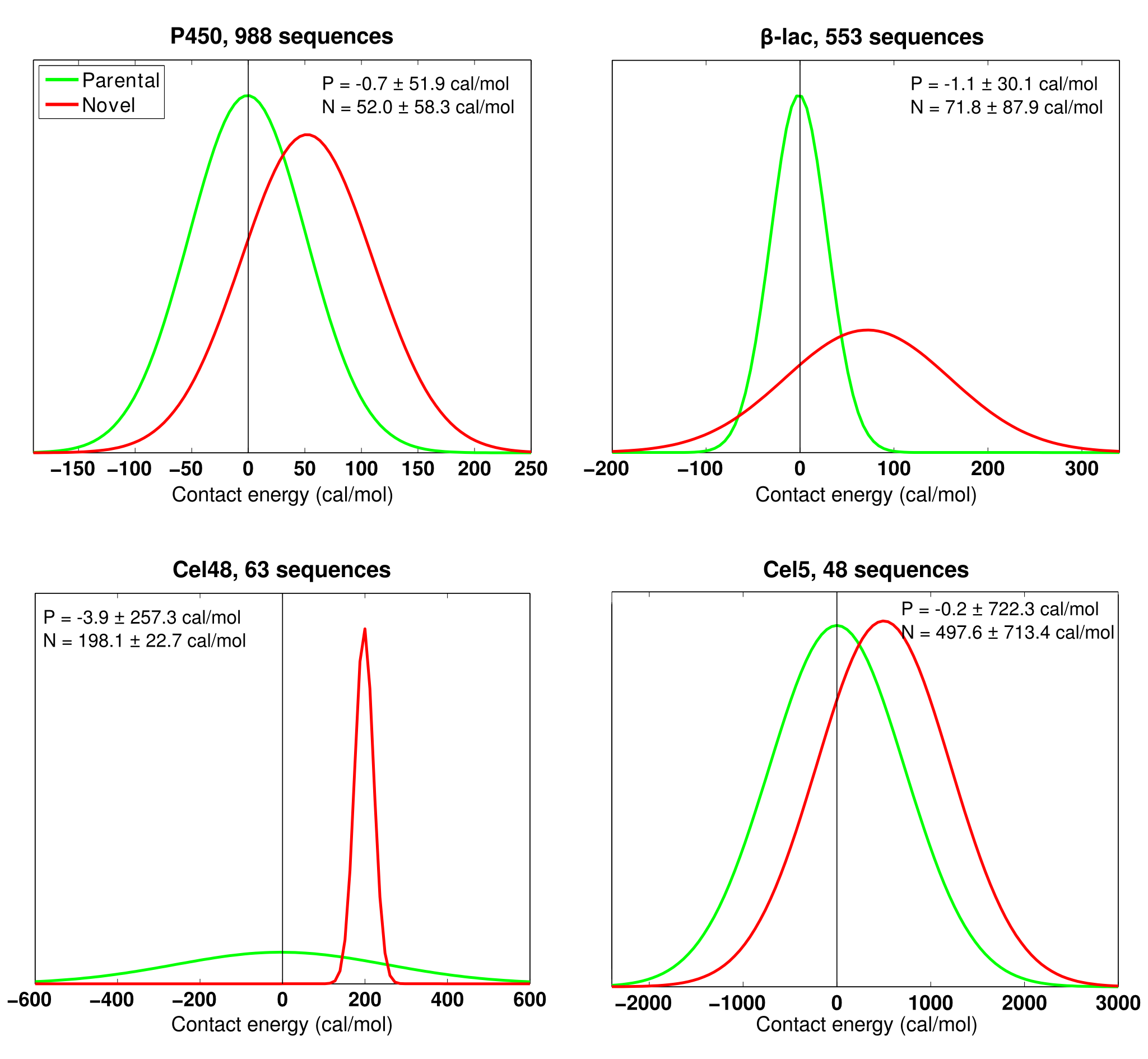

Supplement: Figure S1 — Estimation of contact parameters on other recombination libraries. The parental and novel contact parameters () were estimated on four binary functional status data sets. The number of sequences in each data set are indicated in the plot titles. The estimated parameters are reported as the mean one standard deviation, and the associated Gaussian probability density functions are plotted. The two largest data sets (P450 and -lactamase) give very similar parameter estimates, while all data sets provide the same qualitative relationships among parameters. Within all four parameter sets, we see the mean of parental contacts is slightly favorable and novel contacts are significantly deleterious. The means of these two distributions are separated by approximately one standard deviation, indicating it is relatively common for parental contacts to be as deleterious as novel contacts, and vice versa. (TIFF) [file pcbi.1002713.s001.tif]
