# Supplementary material for: Random Field Model Reveals Structure of the Protein Recombinational Landscape
Source: PLoS Comput Biol. 2012 Oct 4;8(10):e1002713. doi: 10.1371/journal.pcbi.1002713 (PMC3464211; doi:10.1371/journal.pcbi.1002713)
Supplement: Text S1 — Derivation of a library's expected variance and a chimera's additive energy component. A detailed description of how the expected value of a library's variance and the additive component of a chimera's energy are calculated. (PDF) [file pcbi.1002713.s002.pdf]

Random Field Model Reveals Structure of the Protein Recombinational Landscape - Text S1

Philip A. Romero[1], Frances H. Arnold[1,*],
1 Division of Chemistry and Chemical Engineering, California Institute of Technology, Pasadena, CA, USA
* E-mail: frances@cheme.caltech.edu

Expected value of the library variance

Consider a recombination library L, generated by recombining b sequence fragments from p parental sequences. From the definition of the library mean M_L (main text equation 8), the expected value of the library mean within the random field is

$$\mathrm{E}[M_L] = \frac{1}{p^b} \sum_{\mathbf{c} \in L} \mathrm{E}[\mathcal{E}_{\mathbf{c}}] \tag{1}$$

and the variance of the library mean is

$$\mathrm{Var}[M_L] = \frac{1}{p^{2b}} \sum_{\mathbf{c1} \in L} \sum_{\mathbf{c2} \in L} \mathrm{Cov}[\mathcal{E}_{\mathbf{c1}}, \mathcal{E}_{\mathbf{c2}}], \tag{2}$$

where the expected value of the random field $\mathrm{E}[\mathcal{E}_{\mathbf{c}}]$ is defined in main text equation 6, and the covariance within the random field $\mathrm{Cov}[\mathcal{E}_{\mathbf{c1}}, \mathcal{E}_{\mathbf{c2}}]$ is defined in main text equation 7.

Similarly, the expected value of the library variance V_L (main text equation 9) is given by

$$\mathrm{E}[V_L] = \frac{1}{p^b} \sum_{\mathbf{c} \in L} \mathrm{E}\left[(\mathcal{E}_{\mathbf{c}} - M_L)^2\right]) \tag{3}$$

which can be expanded to

$$\mathrm{E}[V_L] = \frac{1}{p^b} \sum_{\mathbf{c} \in L} \left[(\mathrm{E}[\mathcal{E}_{\mathbf{c}}] - \mathrm{E}[M_L])^2 + \mathrm{Var}[\mathcal{E}_{\mathbf{c}}] + \mathrm{Var}[M_L] - 2\,\mathrm{Cov}[\mathcal{E}_{\mathbf{c}}, M_L]\right] \tag{4}$$

where $\mathrm{Var}[\mathcal{E}_{\mathbf{c}}] = \mathrm{Cov}[\mathcal{E}_{\mathbf{c}}, \mathcal{E}_{\mathbf{c}}]$ and

$$\mathrm{Cov}[\mathcal{E}_{\mathbf{c}}, M_L] = -\,\mathrm{E}[\mathcal{E}_{\mathbf{c}}]\,\mathrm{E}[M_L] + \frac{1}{p^b} \sum_{\mathbf{c2} \in L} \left(\mathrm{E}[\mathcal{E}_{\mathbf{c}}]\,\mathrm{E}[\mathcal{E}_{\mathbf{c2}}] + \mathrm{Cov}[\mathcal{E}_{\mathbf{c}}, \mathcal{E}_{\mathbf{c2}}]\right). \tag{5}$$

From this, we can substitute equations 1, 2, and 5 into equation 4 to get an expression for the expected value of the library variance.

Additive component of a chimera's energy

An additive energy function can be defined by considering how individual mutations contribute to variation in the library. Depending on its structural context, a mutation's effect may be constant or varied throughout the library. A chimera's additive energy, which accounts for purely additive and averaged epistatic effects, is given by

$$E_{A,\mathbf{c}} = \sum_i b^i_{\mathbf{c},P}\ \varepsilon^i_P + \sum_i b^i_{\mathbf{c},N}\ \varepsilon^i_N, \tag{6}$$

where $b^i_{\mathbf{c},P}$ and $b^i_{\mathbf{c},N}$ specify how the energy terms ε^i_P and ε^i_N contribute to additive energy of chimera $\mathbf{c}$. The b's are analogous to the a's from main text equation 1. However their values are not binary but rather are determined by the average contribution that an interaction makes to the library. For an interaction i between positions p^i_1 and p^i_2, $b^i_{\mathbf{c},P}$ (and equivalently $b^i_{\mathbf{c},N}$) is given by

$$b^i_{\mathbf{c},P} = \begin{cases} a^i_{\mathbf{c},P} & \text{if } p^i_1 \text{ and } p^i_2 \text{ intra-fragment,} \\ f(i|\mathbf{c},p_1) + f(i|\mathbf{c},p_2) - f(i) & \text{if } p^i_1 \text{ and } p^i_2 \text{ inter-fragment,} \end{cases} \tag{7}$$

where $f(i)$ is the frequency of interaction i within the entire library L, $f(i|\mathbf{c},p_1)$ is the frequency of interaction i in the subset of the library that has the same residue at position p^i_1 as chimera $\mathbf{c}$, and $f(i|\mathbf{c},p_2)$ is the frequency of interaction i in the subset of the library that has the same residue at position p^i_2 as chimera $\mathbf{c}$. This additive energy can be used to calculate a library's additive variance V_A.
